# An update: is there a relationship between *H. pylori* infection and nonalcoholic fatty liver disease? why is this subject of interest?

**DOI:** 10.3389/fcimb.2023.1282956

**Published:** 2023-12-08

**Authors:** Xingcen Chen, Ruyi Peng, Dongzi Peng, Jia Xiao, Deliang Liu, Rong Li

**Affiliations:** ^1^ Department of Gastroenterology, the Second Xiangya Hospital of Central South University, Changsha, Hunan, China; ^2^ Research Center of Digestive Diseases, Central South University, Changsha, Hunan, China; ^3^ Clinical Research Center, Digestive Diseases of Hunan Province, Changsha, Hunan, China

**Keywords:** Helicobacter pylori, extragastric disease, nonalcoholic fatty liver disease, meta-analysis, pathogenesis

## Abstract

*Helicobacter pylori* (*H. pylori*) infection is thought to impact various extragastric diseases, including nonalcoholic fatty liver disease (NAFLD), the most common chronic liver disease. Meanwhile, the pathogenesis of NAFLD needs further research, and effective treatment for this disease remains elusive. In this mini-review, we enumerate and ponder on the evidence demonstrating an association between *H. pylori* infection and NAFLD. Primarily, we delve into high-quality meta-analyses and clinical randomized controlled trials focusing on the association studies between the two. We also discuss clinical studies that present opposite conclusions. In addition, we propose a mechanism through which *H. pylori* infection aggravates NAFLD: inflammatory cytokines and adipocytokines, insulin resistance, lipid metabolism, intestinal barrier and microbiota, *H. pylori* outer membrane vesicles and *H. pylori*-infected cell-extracellular vesicles. This mini-review aims to further explore NAFLD pathogenesis and extragastric disease mechanisms caused by *H. pylori* infection.

## 1 Introduction

*Helicobacter pylori* (*H. pylori*) is a gram-negative bacillus that colonizes the human stomach. It is microaerophilic and has the ability to metabolize urea into ammonia and carbon dioxide. *H. pylori* is transmitted through the oral-oral and fecal-oral routes, and it infects approximately 4.4 billion people worldwide, with a prevalence rate of 44.3% (95% CI: 40.9-47.7) (Hooi et al., 2017; Sjomina et al., 2018; Zamani et al., 2018). An epidemiological survey based on family units revealed that the prevalence of *H. pylori* in China was approximately 40.66%, 43.45% in adults, and 20.55% in children and adolescents (Zhou et al., 2023). Multiple studies have substantiated that *H. pylori* infection serves as the initiating factor in the progression of chronic gastritis to gastric cancer (Correa, 1992; Uemura et al., 2001; Kusters et al., 2006). In addition to peptic ulcers, gastritis, gastric cancer, and other gastric diseases, a variety of extragastric diseases, such as stroke, Alzheimer’s disease, and nonalcoholic fatty liver disease (NAFLD), are closely related to *H. pylori* infection (Santos et al., 2020).

NAFLD refers to a spectrum of diseases, including simple hepatocellular steatosis, nonalcoholic steatohepatitis (NASH), NASH-associated cirrhosis, and hepatocellular carcinoma. With changes in diet and lifestyle, NAFLD is the the most common chronic liver disease (Chalasani et al., 2018; Younossi et al., 2020). Moreover, in the United States, NAFLD is the second leading cause of liver transplantation after alcoholic liver disease (Kim et al., 2018). The overall global prevalence of NAFLD was estimated to be approximately 29.1% (95% CI: 26.8-31.5), with the highest prevalence in Latin America (44.4%) and the lowest in Western Europe (24.6%). Moreover, the global prevalence of NAFLD is progressively increasing, rising from 24.4% in 1991-2006 to 36.0% in 2016-2020 (Henry et al., 2022). However, the pathogenesis of NAFLD remains unknown. The *multiple-hit* pathogenesis reviewed by Buzzetti et al. is widely accepted in academia and includes insulin resistance (IR), hormones secreted fromadipose tissue, nutritional factors, and gut microbiota (Buzzetti et al., 2016). No agents have been approved for the clinical treatment of NAFLD, and their treatment options rely mainly on weight loss through dietary modification and physical exercise (Wang and Malhi, 2018). Therefore, it is necessary to correctly discern the pathogenesis of NAFLD and propose targeted treatment options for NAFLD.

Since the initial report of *H. pylori* DNA being detected in the liver of NAFLD patients (Cindoruk et al., 2008), numerous studies have investigated the relationship between *H. pylori* infection and NAFLD (Kim et al., 2017; He et al., 2018; Yu et al., 2022). Based on the pandemic and the rate of *H. pylori* infection and NAFLD worldwide, a large proportion of patients have comorbid diseases. Despite the increasing severity of antibiotic-resistant forms of *H. pylori*, the eradication rate with multiple first-line treatment regimens remains more than 80% (Rokkas et al., 2021). Compared with NAFLD, for which no effective medical therapy is currently available, *H. pylori* can be eradicated in a large proportion of patients with comorbidities. In that case, it can delay or improve the progression of NAFLD and maximize the benefit of patients with comorbid conditions while greatly relieving the disease burden of NAFLD. However, there remains controversy about whether *H. pylori* infection is clinically associated with NAFLD, with some extensive multicenter clinical studies suggesting an association (Sumida et al., 2015; Kim et al., 2017; Doulberis et al., 2020) and others suggesting no association (Jamali et al., 2013; Okushin et al., 2015; Baeg et al., 2016). Even if they are relevant, mechanistic studies are needed to conduct research on how *H. pylori* infection impacts NAFLD. This mini-review intends to review the association between *H. pylori* and NAFLD and propose a hypothesis according to the summarized literature: *H. pylori* infection may exacerbate the development of NAFLD. Moreover, the authors explore the mechanism of the occurrence and development of NAFLD and the mechanism of extragastric diseases caused by *H. pylori.*

## 2 Preclinical studies

Basic research on *H. pylori* infection and NAFLD is rare, and no precise mechanism has been found. He et al. (He et al., 2018) established a mouse model of *H. pylori* infection, fed a high-fat diet (HFD) and a chow diet for six months, which showed that HFD plus *H. pylori*-infected mice had significantly increased abdominal circumference, fasting blood glucose (FBG), low-density lipoprotein cholesterol (LDL-C), and alanine aminotransferase (ALT) compared with HFD controls, and showed more severe hepatic steatosis, which was consistent with our hypothesis. Liver fibrosis is a progressive manifestation of NAFLD (Friedman et al., 2018). *H. pylori* infection has demonstrated to promote CCl_4_-induced liver fibrosis in animal models (Goo et al., 2009), and that the proinflammatory signaling pathways may occur through transforming growth factor-beta1 (TGF-β1) (Ki et al., 2010).

The progress of experimental research on the association between *H. pylori* infection and NAFLD has been slow, which could be attributed to the following factors: (1) challenges in mimicking the complex hepatic physiological environment in cellular and molecular experiments; (2) *H. pylori* infection does not promote NAFLD through direct pathways; and (3) *H. pylori* infection is not associated with NAFLD. More experimental studies in line with human physiology, such as hepatic organoids, are needed to demonstrate whether *H. pylori* infection is associated with NAFLD.

## 3 Clinical studies

### 3.1 Meta-analyses revealed a positive correlation between *H. pylori* infection and NAFLD

**Table 1** summarizes nine meta-analyses of clinical research on *H. pylori* infection with NAFLD (Wijarnpreecha et al., 2018; Liu et al., 2019; Mantovani et al., 2019; Ning et al., 2019; Zhou et al., 2019; Wei and Ding, 2021; Heydari et al., 2022; Ma et al., 2022; Xu et al., 2023). These studies included a minimum of 38,622 and a maximum of 218,573 participants. Most clinical studies included in these meta-analyses were conducted in the Asian region (China, Japan, and South Korea), and a few involved Europe, the United States, and Egypt. The results of the subgroup analysis suggested that *H. pylori* infection was stable and associated with NAFLD in Asian regions (*P* < 0.01), and there were no uniform results in other regions due to different inclusion studies in each meta-analysis (Mantovani et al., 2019; Ning et al., 2019; Zhou et al., 2019; Wei and Ding, 2021; Xu et al., 2023). This may be due to differences in the cytotoxin-associated gene A (Cag A) status of *H. pylori* and strain virulence between Asia and other regions (Yamaoka, 2009; Park et al., 2018). The heterogeneity of these meta-analyses was generally high, which may be related to the differences in the region, population, and methods of diagnosis included in the study. Only one meta-analysis had publication bias (Ning et al., 2019), which could be due to the small sample size of the studies. After removing two studies with small sample sizes, publication bias was eliminated, and *H. pylori* infection remained significantly associated with NAFLD. All meta-analyses demonstrated a positive association between *H. pylori* infection and NAFLD. The odds ratio (OR) ranged from 1.14 to 1.53, which meant that the proportion of *H. pylori* infection in NAFLD patients was 1.14 - 1.53 times higher than that in their non-NAFLD counterparts. We hypothesize that *H. pylori* infection alone may have difficulty causing NAFLD, but *H. pylori* infection combined with a fast food diet and lifestyle disorders may exacerbate NAFLD levels.

**Table 1:** Meta-analyses of the association between *H. pylori* infection and NAFLD.

Reference	Included studies (N)	Participants (N)	Main results	Odds ratio (OR)	Heterogeneity (I^2^)	Publication bias
**Wijarnpreecha K, 2018** (Wijarnpreecha et al., 2018)	6 (5 cross-sectional, 1 case-control)	38,622	A significantly increased risk of NAFLD among patients with *H. pylori* infection	1.21 (95% CI: 1.07–1.37)	49.00%	Indeterminant (evaluated only by Funnel plot)
**Mantovani A, 2019** (Mantovani et al., 2019)	13 (2 cohort, 2 case-control, 9 cross-sectional)	81,162	*H. pylori* infection is associated with mildly increased risk of NAFLD	1.14 (95% CI: 1.05–1.23)	59.60%	No
**Zhou BG, 2019** (Zhou et al., 2019)	15 (2 cohort, 2 case-control, 11 cross-sectional)	97,228	A positive association between *H. pylori* infection and the risk of NAFLD	1.19 (95% CI: 1.11–1.29)	66.00%	No
**Ning L, 2019** (Ning et al., 2019)	12 (2 cohort, 2 case-control, 8 cross-sectional)	87,747	*H. pylori* infection was associated with the development of NAFLD	1.36 (95% CI: 1.22–1.53)	89.60%	Yes
**Liu R, 2019** (Liu et al., 2019)	21 (2 cohort, 2 case-control, 17 cross-sectional)	145,091	*H. pylori* infection is one of the factors that promotes the progression of NAFLD	1.53 (95% CI: 1.34 - 1.75)	95.60%	No
**Wei L, 2021** (Wei and Ding, 2021)	17 (2 cohort, 1 case-control, 14 cross-sectional)	91,958	A positive association between *H. pylori* infection and the risk of NAFLD	1.38 (95% CI: 1.23–1.55)	86.80%	No
**Ma Z, 2022** (Ma et al., 2022)	25 (2 cohort, 6 case-control, 17 cross-sectional)	107,306	*H. pylori* infection was associated with an increased risk of NAFLD	1.30 (95% CI: 1.13–1.49) (Asian); 1.42 (95% CI: 1.04–1.94) (non-Asian)	94.30% (Asian);44.90% (non-Asian)	No
**Heydari K, 2022** (Heydari et al., 2022)	22 (2 cohort, 2 case-control, 18 cross-sectional)	117,117	*H. pylori* infection increases the risk of developing NAFLD	1.22 (95% CI: 1.09-1.35)	NA	NA
**Xu G, 2023** (Xu et al., 2023)	34 (4 cohort, 3 case-control, 27 cross-sectional)	218,573	*H. pylori* infection is associated with NAFLD	1.26 (95% CI: 1.17–1.36)	88.70%	No

NAFLD, non alcoholic fatty liver disease; CI, Confidence interval; NA, not available.

The meta-analyses performed subgroup analyses according to race, region, diagnostic methods, and different study types to make the results more robust and credible and to reduce overall heterogeneity. However, most included studies were cross-sectional and could not illustrate the causal relationship between *H. pylori* and NAFLD. Second, correcting the effects of confounding factors such as hygiene level, dietary habits, physiological activity, and genetics from the clinical data collected by meta-analyses was difficult. With the specification of the NAFLD definition, the nomenclature of new fatty liver disease- metabolic dysfunction-associated steatotic liver disease (MASLD) (Rinella et al., 2023) will provide more accurate and high-quality clinical studies on the relationship between *H. pylori* infection and NAFLD/MASLD.

### 3.2 Dialectical discussion on studies negating the *H pylori*-NAFLD link

The above meta-analyses results suggest that *H. pylori* infection is positively associated with NAFLD. However, some clinical studies are controversial to this conclusion. We collected 13 clinical studies on *H. pylori* infection that had no bearing on NAFLD, including one bidirectional Mendelian randomization (MR) study (Liu et al., 2022), two clinical trials (Jamali et al., 2013; Polyzos et al., 2014), and ten cross-sectional studies (Okushin et al., 2015; Baeg et al., 2016; Cai et al., 2018; Fan et al., 2018; Kang et al., 2018; Lu et al., 2018; Han et al., 2021; Rahman et al., 2021; Wang et al., 2022a; Wernly et al., 2022), as shown in **Table 2**. Then, we provide a detailed dialectical discussion of clinical trials on studies needing revised or improved designs. A detailed discussion of the clinical randomized controlled trials (RCTs) by Jamali et al. (Jamali et al., 2013) and Polyzos et al. (Polyzos et al., 2014) is provided in Section 2.3.

**Table 2:** Clinical studies on *H. pylori* infection is not associated with NAFLD.

Reference	Region	Participants (N)	Research type	Main results	Odds ratio (OR)
**Liu Y, 2022** (Liu et al., 2022)	Europe	NA	A bidirectional Mendelian randomization study	No evidence for a causal link between *H. pylori* infection and NAFLD	1.05 (95% CI: 0.78-1.41)
**Jamali R, 2013 (** Jamali et al., 2013)	Iran	100 (50:50)	Randomized open-label clinical trial	*H. pylori* eradication might not affect LFC, LFT, lipid profile, and insulin resistance in dyspeptic NAFLD patients	NA
**Polyzos SA, 2014** (Polyzos et al., 2014)	Greece	13 (6:7)	prospective clinical research	*H. pylori* eradication had no long-term effect on hepatic steatosis.	NA
**Okushin K, 2015** (Okushin et al., 2015)	Japan	1,802	cross-sectional study	*H. pylori* infection status did not show significant association with NAFLD	1.13 (95% CI: 0.99-1.28)
**Baeg MK, 2016 (Baeg et al., 2016)**	Republic of Korea	3,663	cross-sectional study	*H. pylori* infection is not a risk factor for NAFLD	1.13 (95% CI: 0.97-1.31)
**Kang SJ, 2018** (Kang et al., 2018)	the United States	5,404	cross-sectional study	CagA positive *H. pylori* group did not demonstrate an association with NAFLD; CagA negative *H. pylori* group had a significant association with NAFLD	1.05 (95% CI: 0.86-1.18) (cagA positive) 1.30 (95% CI: 1.01-1.67) (cagA negative)
**Lu LJ, 2018** (Lu et al., 2018)	China	1,867	cross-sectional study	participants with NAFLD had no statistically significant differences of *H. pylori* infection than those without NAFLD	1.13 (95% CI: 0.92-1.39)
**Fan N, 2018** (Fan et al., 2018)	China	21,456	cross-sectional study	*H. pylori* infection is not independently associated with the risk of NAFLD	1.00 (95% CI: 0.70-1.30)
**Cai O, 2018 (** Cai et al., 2018)	China	2,051	cross-sectional study	*H. pylori* infection does not increase the NAFLD prevalence rate or to be associated with, or a risk factor for, NAFLD.	0.94 (95% CI: 0.70-1.27)
**Han YM, 2021** (Han et al., 2021)	Republic of Korea	1,784	cross-sectional study	*H. pylori* seropositivity was not associated with CAP-defined NAFLD	0.96 (95% CI: 0.78-1.19)
**Rahman MM, 2021** (Rahman et al., 2021)	Bangladesh	767	cross-sectional study	There was no relationship observed between *H. pylori* seroprevalence and NAFLD.	1.50 (95% CI: 0.94- 2.39)
**Wang W, 2022** (Wang et al., 2022a)	China	71,633	cross-sectional study	*H. pylori* infection was not an independent risk factor for NAFLD.	1.02 (95% CI: 0.97-1.08)
**Wernly S, 2022** (Wernly et al., 2022)	Austria	5,338	cross-sectional study	No independent association was found between *H. pylori* infection and NAFLD.	0.96 (95%CI: 0.82-1.13)

NAFLD, non alcoholic fatty liver disease; CI, Confidence interval; NA, not available; LFC, liver fat content; LFT, liver function tests; CAP, controlled attenuation parameter; Cag A, cytotoxin-associated gene A.

MR, which uses single nucleotide polymorphisms as instrumental variables to investigate the causal relationship between exposure factors and disease, have been widely used in epidemiological causal inferences in recent years (Davey Smith and Hemani, 2014). Liu et al. (Liu et al., 2022) showed no causal link between *H. pylori* infection and NAFLD. Moreover, *H. pylori* infection was not significantly associated with triglycerides (TG), LDL-C, high-density lipoprotein cholesterol (HDL-C), or FBG. However, in this study, *H. pylori* infection diagnosis was based on serological testing and only involved European participants, which could have made the results potentially over-evaluated.

The cross-sectional study results were quite different. The lower 95% CI of the OR for some studies (Okushin et al., 2015; Baeg et al., 2016; Rahman et al., 2021; Wang et al., 2022a) was very close to 1, suggesting that the statistical results have questionable reliability in practice. This may be due to (1) different *H. pylori* strains circulating in various regions; (2) *H. pylori* infection not being diagnosed uniformly, such as ^13^C- urea breath test and serum *H. pylori* antibody detection; (3) NAFLD being diagnosed differently, such as ultrasonography and hepatic steatosis index (HSI) and NAFLD liver fat score (NAFLD-LFS); and (4) differences in race and living routine between regions. Fan et al.(Fan et al., 2018) showed a significant association between *H. pylori* infection and NAFLD after adjusting for age and sex (OR = 1.1, 95% CI: 1.0 - 1.1, *P* = 0.004). Nevertheless, after adjusting for body mass index (BMI) and systolic and diastolic blood pressure, there was no significant relationship between them (OR = 0.9, 95% CI: 0.9 - 1.0, *P* = 0.097). Finally, fasting plasma glucose, hemoglobin A1C (HbA1c), triglycerides, total cholesterol, HDL-C, LDL-C, and serum creatinine were adjusted (OR = 1.0, 95% CI: 0.7 - 1.3, *P* = 0.753). After adjusting for the metabolic index, *H. pylori* infection was not associated with NAFLD, which suggested that *H. pylori* infection may lead to metabolic disturbances rather than directly causing NAFLD. Our cross-sectional study of 16,942 participants also presents a significant link between *H. pylori* infection and metabolic index in humans (unpublished data), consistent with existing findings (Buzás, 2014).

### 3.3 Clinical RCTs on *H. pylori* eradication in *H. pylori*-positive NAFLD patients

Clinical RCTs are the gold standard for assessing the theoretical efficacy of clinical interventions. We collected recent RCTs on *H. pylori* eradication therapy in *H. pylori*-positive NAFLD patients (Jamali et al., 2013; Polyzos et al., 2014; Abdel-Razik et al., 2018; Maharshi et al., 2020; Yu et al., 2022). According to clinical RCTs, we speculated that eradicating *H. pylori* in *H. pylori* -positive NAFLD patients contributes to improving metabolic parameters.

The first randomized open-label clinical trial (Jamali et al., 2013) finally enrolled 100 patients with NAFLD randomly divided into a lifestyle modification group and a lifestyle modification plus *H. pylori* eradication group. After six months, it was found that liver fat content, liver function tests, lipid profile, and IR were improved in both groups compared with baseline levels. Nonetheless, there was no statistically significant difference between the two groups. Notably, the results of a recent randomized controlled trial of *H. pylori* eradication in NAFLD patients were the opposite. Yu et al. (Yu et al., 2022) enrolled 191 NAFLD patients with *H. pylori* infection and randomly divided them into untreated (health education and lifestyle guidance) and treated (health education and lifestyle guidance plus 14 days of *H. pylori* quadruple therapy) groups. One year later, the patient’s metabolic index and FibroScan controlled attenuation parameter (CAP) values improved compared to those before treatment. The metabolic index [FBG, HbA1c (%), homeostatic model assessment of insulin resistance (HOMA-IR), TG, and BMI], CAP value, and inflammatory parameters [white blood cells, high-sensitivity C-reactive protein, interleukin-6 (IL-6), tumor necrosis factor-alpha (TNF-α)] of the treated group were significantly improved compared with those of the untreated group. These two prospective studies came to a paradoxical conclusion, which may be mainly due to differences in inclusion criteria and follow-up times. Jamali et al. included NAFLD patients with ALT and AST greater than the upper limit of normal (ULN) who developed dyspeptic symptoms. Yu et al. included NAFLD patients with ALT and AST < 2 times the ULN and no gastrointestinal disease symptoms. In other words, Jamali et al. included patients with moderate and severe NAFLD; Yu et al. included patients with mild NAFLD. There was no difference in *H. pylori* eradication between the two groups in the former and a significant improvement between the two groups in the latter, illustrating the necessity of early eradication of *H. pylori* in NAFLD patients with *H. pylori* infection. Polyzos et al. (Polyzos et al., 2014) recruited 13 patients with biopsy-proven NAFLD who were divided into *H. pylori* (+) and *H. pylori* (−) groups according to whether they had *H. pylori* infection, both of whom received standard instructions for diet and exercise, and the *H. pylori* (+) group received *H. pylori* eradication therapy. Hepatic steatosis, HSENSI [Homocysteine, serum glutamic oxaloacetic transaminase, Erythrocyte sedimentation rate, and Nonalcoholic Steatohepatitis Index] were assessed twelve months later. The results indicated that *H. pylori* eradication had no long-term effect on magnetic resonance imaging -assessed hepatic steatosis. However, they suggested a trend toward improved NAFLD fibrosis score and HSENSI with *H. pylori* eradication. Interestingly, a retrospective study by the Polyzos team (Doulberis et al., 2020) suggested that active *H. pylori* infection was significantly associated with liver function, HOMA-IR, and liver fibrosis stage. The inclusion criteria for this study were patients with NAFLD demonstrated by liver biopsy and divided into *H. pylori* (+) and *H. pylori* (−) groups according to the presence or absence of *H. pylori* infection demonstrated by gastric biopsy. The findings of the same team seem paradoxical, probably because prospective studies included insufficient participants.

Abdel-Razik et al. (Abdel-Razik et al., 2018) followed 369 NAFLD patients (171 *H. pylori*-positive and 198 *H. pylori-*negative) for 24 months. They found that *H. pylori* eradication significantly reduced IR, lipid profile, HSI, and NAFLD-LFS and increased HDL. Maharshi et al. (Maharshi et al., 2020) followed 64 NAFLD patients (36 *H. pylori*-positive and 28 *H. pylori*-negative) for six months and found that *H. pylori* eradication improved hepatic steatosis and metabolic parameters. These findings also coincide with the results of another prospective RCT (Gen et al., 2010): beneficial impacts of *H. pylori* eradication therapy on IR, atherogenic lipid abnormalities, and low-grade inflammation.

## 4 The possible mechanism of *H. pylori* infection exacerbating NAFLD

**Figure 1:**
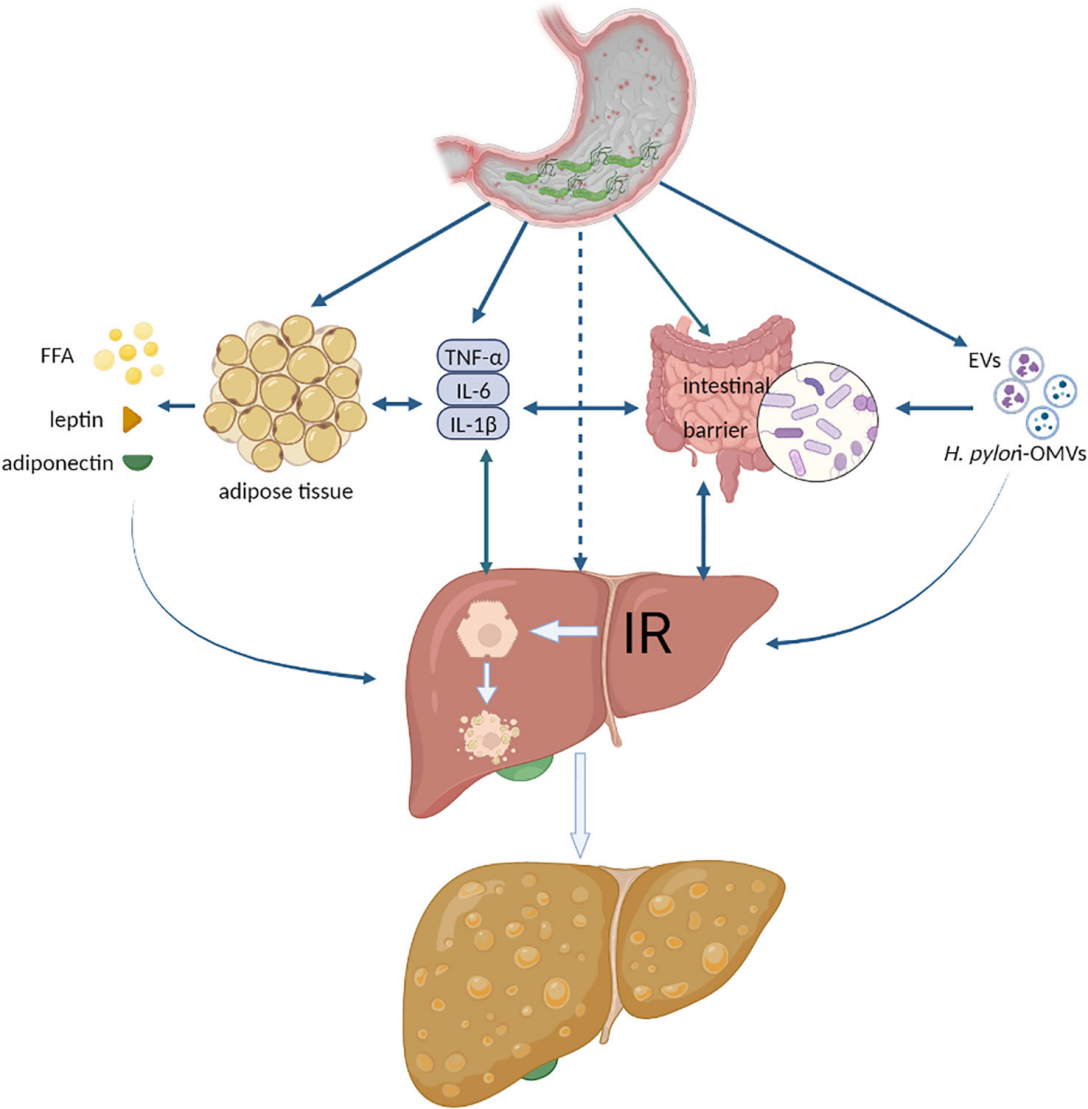
The possible mechanisms of *H. pylori* infection exacerbating NAFLD. FFA, free fatty acid; IR, insulin resistance; EVs, extracellular vesicles; *H. pylori-*OMVs, *H. pylori-* outer membrane vesicles. Authors hypothesize that systemic low-grade systemic inflammation caused by *H. pylori* infection aggravates NAFLD. Various inflammatory cytokines act directly or indirectly on adipose tissue, liver, and intestine to trigger or aggravate IR, disrupt the intestinal barrier, lead to hepatocyte steatosis, or activate liver fibrosis. Another possible mechanism is extracellular vesicles released by *H. pylori*-infected host cells or outer membrane vesicles secreted by *H. pylori*, which act directly with the liver and promote the development of NAFLD.

Currently, there is no direct experimental mechanistic evidence that *H. pylori* infection impacts NAFLD, and the primary explanation focuses on IR, inflammatory cytokines or adipocytokines, lipid metabolism, and the intestinal barrier (Li et al., 2013; Cheng et al., 2017; Doulberis et al., 2021). We summarize the latest experimental evidence on the effects of *H. pylori* on the above factors and propose a new pathway: extracellular vesicles (EVs) or *H. pylori* outer membrane vesicles (*H. pylori-*OMV*s*), as shown in **Figure 1**. It should be noted that these possible mechanisms do not act independently on the development of NAFLD, such as multiple inflammatory cytokines that can also be involved in IR, lipid metabolism disorders, and intestinal barrier dysfunction. These factors are jointly engaged in multiple hits in NAFLD.

### 4.1 Inflammatory cytokines and adipocytokines

Inflammatory cytokines play a critical role in the pathology of *H. pylori* infection and NAFLD. In patients with persistent *H. pylori* infection, the body may lead to a chronic low-grade inflammation state and increased levels of the NOD-like receptor protein 3 (NLRP3) inflammasome (Pérez-Figueroa et al., 2016) and inflammatory cytokines, such as interleukin-1beta (IL-1β), IL-6, and TNF-α (Buzás, 2014). Inflammasomes and inflammatory cytokines are secreted by *H. pylori*-infected gastric epithelial cells and mucosal and circulating monocytes (Algood and Cover, 2006), reaching the liver via the circulatory system. NLRP3 and IL-1β participat in the whole process of liver inflammation, including IR and liver fibrosis (Tilg et al., 2016). IL-1β is directly involved in IL-1β/TNF-induced hepatocyte necrosis (Shen et al., 2020), promoting hepatic steatosis (Stienstra et al., 2010; Negrin et al., 2014), with positive feedback amplification of inflammation-induced IL-1β and TNF-α (Petrasek et al., 2012). In addition, researchers have found that IL-1β inhibits the fibroblast growth factor 21 (FGF 21) coreceptor beta-Klotho (KLB) to suppress FGF21 physiological effects (Zhao et al., 2016), which is crucial in regulating hepatic lipid metabolism and anti-inflammation (Xu et al., 2009). Mitsuyoshi et al. found that NLRP3, procaspase-1, IL-1β, and IL-18 mRNA levels were significantly increased in the livers of NAFLD patients compared to healthy controls (Mitsuyoshi et al., 2017). Accordingly, choline-deficient amino acid-defined diet-induced hepatomegaly, liver inflammation, and fibrosis were significantly ameliorated in Nlrp3 knockout mice compared with wild-type mice (Wree et al., 2014). Similar to IL-1β and NLRP3, TNF-α, and IL-6 play a direct or indirect role in the progression of NAFLD. TNF-α directly increases the expression of mast cell proteinase 1, Tgfb1, and tissue inhibitor of metalloproteinase 1 in hepatocytes, participating in hepatic lipid metabolism, inflammation, and liver fibrosis processes (Kakino et al., 2018). It is well-known that TNF-α also phosphorylates the serine of insulin receptor substrate-1 (IRS-1) to induce IR (Hotamisligil et al., 1996). Studies have demonstrated that IL-6 is involved in NASH progression through the IL-6/signal transducer and activator of transcription 3 (STAT3) signaling pathway (Cai et al., 2016; Li et al., 2022).

Visceral white adipose tissue (WAT), when infiltrated by inflammatory cells, releases adipocytokines (including adiponectin, leptin, and resistin) and inflammatory factors (such as TNF-α and IL-6), which are involved in regulating IR and inflammation via endocrine or paracrine mechanisms (Kershaw and Flier, 2004; Stojsavljević et al., 2014). Adiponectin plays a role in alleviating IR and reducing intrahepatic triglyceride accumulation and is well known as a protective factor for NAFLD. On the one hand, adiponectin activates AMP-activated kinase (AMPK) by binding to adiponectin receptor 2 on the surface of hepatocytes, inhibits acetyl-CoA carboxylase, and decreases malonyl-CoA production, thereby increasing β-oxidation of fatty acids (FAs). On the other hand, adiponectin inhibits glycogenolysis and gluconeogenesis by inhibiting glucose-6-phosphatase and phosphoenolpyruvate carboxyl kinase mRNA expression (Combs and Marliss, 2014). Normally, hepatic leptin activates the phosphatidylinositol-3 kinase/Akt (protein kinase B)/mammalian target of rapamycin (mTOR) pathway mainly by binding to hepatocyte surface leptin receptor b, suppressing hepatic glucose production and improving insulin sensitivity. In addition, leptin can also activate the Janus kinase 2/STAT3 signaling pathway in the liver and regulate the function of suppressors of cytokine signaling 3 (SOSC3) function, a negative feedback regulatory molecule of the leptin receptor signaling pathway (Polyzos et al., 2015). However, the association of *H. pylori* infection with serum adipocytokines remains debated, as clinical observational studies have reached contradictory conclusions. Chen et al. found no significant difference in circulating leptin and adiponectin levels between *H. pylori*-positive and *H. pylori*-negative patients (Chen et al., 2015). Abdel-Razik et al. found a significant decrease in serum leptin and no significant change in adiponectin levels after *H. pylori* eradication therapy (Abdel-Razik et al., 2018), while Ando et al. found a significant increase in serum adiponectin levels after *H. pylori* eradication therapy (Ando et al., 2013). Future large-scale, prospective studies are needed to find specific links between *H. pylori* infection and adipocytokines.

### 4.2 Insulin resistance

IR, a central cause of NAFLD development, plays a significant role in hepatic triglyceride deposition, the inflammatory response, and hepatic fibrosis progression (Watt et al., 2019). Numerous studies have shown that *H. pylori* infection is an independent risk factor for IR. A cross-sectional study involving 1107 participants found that IR patients had a significantly higher prevalence rate of *H. pylori* infection than non-IR patients, even after adjusting for sex, age, BMI, waist circumference, visceral and subcutaneous adipose tissue, smoking status, alcohol consumption, dietary habits, and physical activity (Gunji et al., 2009). In *H. pylori*-infected patients, fasting glucose, fasting insulin, HbA1c, and HOMA-IR values decreased significantly after *H. pylori* eradication compared with those before treatment (Dogan et al., 2015). In addition, multiple meta-analyses have also indicated a link between *H. pylori* infection and IR (Polyzos et al., 2011; Azami et al., 2021). Animal studies have shown that HFD-fed mice with *H. pylori* infection developed more severe IR than HFD-fed mice alone, and mice fed a HFD for 12 weeks plus *H. pylori* infection were obese similar to mice fed a HFD for 24 weeks (He et al., 2016). Further studies revealed that *H. pylori* infection increases the expression of the inflammation-related transcription factor c-Jun. C-Jun can bind to the promoter region of the miR-203 gene to inhibit miR-203 expression, an inhibitor of the insulin negative feedback regulator SOCS3, and ultimately promote hepatic IR through the c-Jun/miR-203/SOCS3 pathway (Zhou et al., 2015).

### 4.3 Lipid metabolism

Hepatocyte steatosis is the primary pathological manifestation of NAFLD; its essence is lipid metabolism disorder in hepatocytes (Powell et al., 2021). Clinical studies have suggested that *H. pylori* infection affects lipid metabolism (Buzás, 2014; Watanabe et al., 2021). A large-cohort propensity score-matched analysis revealed that eradicating *H. pylori* could alleviate the deterioration of lipid metabolism but not return to uninfected levels. Specifically, HDL-C continued to decrease, LDL-C continued to rise in *H. pylori*-infected patients after *H. pylori* eradication therapy, and their lipid changes were significantly greater than those in participants with persistent *H. pylori*-negative status and significantly smaller than those in patients with persistent *H. pylori*-positive status (Wang et al., 2022b).

Increased triglyceride synthesis [fatty acid uptake, *de novo* lipogenesis (DNL)] and decreased consumption [fatty acid β-oxidation, very low-density lipoprotein (VLDL) transport] in the liver are the leading causes of triglyceride deposition (Kawano and Cohen, 2013). Donnelly et al. (Donnelly et al., 2005) demonstrated hepatic triglyceride deposition in NAFLD patients, 59% from plasma nonesterified fatty acids [also called free fatty acids (FFAs)], 26% from *de novo* lipogenesis, and 15% from the diet. FFAs in plasma are mainly derived from adipose tissue lipolysis. There is no direct linkage to the evidence that *H. pylori* infection increases adipose tissue lipolysis, whereas chronic systemic inflammation triggered by *H. pylori* infection induces WAT lipolysis (Xu et al., 2003). It has been found that fatty acid synthase and ATP-citrate lyase, critical enzymes of DNL in the gastric mucosa of *H. pylori*-infected patients, are upregulated, which means that DNL in the gastric mucosa of *H. pylori*-infected patients is increased (Chen et al., 2020), but whether *H. pylori* infection directly affects DNL in the liver needs further experimental research. Moreover, *H. pylori* infection affects the intestinal barrier and gut microbiota, affecting diet-derived lipid metabolism (see Section 4.4). VLDL is a carrier for transporting TGs synthesized by the liver to peripheral organs or circulation. When hepatic lipid production is excessive and VLDL transport capacity is not matched, hepatic steatosis and lipotoxicity produced by hepatic triglyceride accumulation. It causes dyslipidemia, on the other hand (Heeren and Scheja, 2021). Circulating VLDL is significantly higher in *H. pylori*-infected patients than in non-infected patients (Işıktaş Sayılar et al., 2015), which may result indirectly from IR caused by *H. pylori* infection or endoplasmic reticulum stress in hepatocytes.

### 4.4 Intestinal barrier and microbiota

Substantial evidence has demonstrated that *H. pylori* infection impacts intestinal barrier function. Under pathological conditions such as hypoxia, inflammatory response, and intestinal microbiota disorders, intestinal bacteria, and their metabolites pass through the damaged intestinal barrier, enter the circulation and participate in the development of NAFLD (Sanduzzi Zamparelli et al., 2016).

A clinical study matching participants’ sex, age, BMI, alcohol consumption, smoking, proton pump inhibitor usage, history of peptic ulcer disease, and dietary habits found that *H. pylori* infection was associated with alterations in the fecal microbiota and increased overall diversity of fecal microbes (Frost et al., 2019). Experimental studies have found that mice fed a HFD plus *H. pylori* infection have increased intestinal abundance of *Helicobacter* and decreased abundance of *Lactobacillus*, with a loss of diversity. Meanwhile, *H. pylori* infection also aggravated HFD-induced hyperglycemia, which could not be restored even with *H. pylori* eradication (Peng et al., 2021). Another study in mice fed a HFD plus *H. pylori* infection found that the expression of tight junction components, such as occludin, zonula occludens-1, and claudin-1, which are important components of the intestinal barrier, was significantly decreased, indicating that *H. pylori* infection directly affects intestinal barrier function (He et al., 2016). Further studies revealed that CagA -containing exosomes increased intestinal permeability by upregulating Claudin-2 expression through activation of CDX2 (Caudal-related homeodomain transcription 2) (Guo et al., 2022). Intestinal barrier dysfunction gives rise to (1) increased intestinal permeability, intestinal bacteria and their metabolites (such as dimethylamine, trimethylamine) and lipopolysaccharide (LPS) in the liver, triggering a liver inflammatory response, hepatocyte damage, and liver fibrosis (Cui et al., 2019); (2) intestinal epithelial cells release inflammatory cytokines to promote NAFLD (Wigg et al., 2001); and (3) intestinal nutrient absorption dysfunction and metabolic substances such as choline deficiency (Spencer et al., 2011).

### 4.5 EVs or *H. pylori*-OMVs

*H. pylori*-OMVs are bilayer membrane spherical vesicles released from *H. pylori* and contain various bacterial elements, such as LPS, outer membrane proteins (OMPs), and virulence proteins [such as CagA and Vacuolating cytotoxin A (Vac A)]. *H. pylori*-OMVs can influence bacterial survival, transmit toxins and virulence factors, and regulate the host immune response and genetic material transfer (Parker and Keenan, 2012). EVs are membranous vesicles secreted by *H. pylori*-infected host cells that maintain intercellular communication, promote inflammatory responses, and manipulate the lesion microenvironment (González et al., 2021). Extracellular vesicles are divided into two categories according to diameter: exosomes and microvesicles, the former of which have been most studied for their biological characteristics.

*H. pylori*-OMVs and *H. pylori*-infected host cell-derived EVs have been found to accelerate the development of various diseases (Chmiela et al., 2018; González et al., 2021; Qiang et al., 2022). CagA-containing exosomes have been demonstrated in the circulation of both CagA-positive *H. pylori*-infected patients and mice (Shimoda et al., 2016; Xia et al., 2020). Exosomes in the blood circulation reach the liver directly, impair endothelial function, activate hepatic Kupffer cells, and promote the hepatic inflammatory response and hepatocyte damage (Kazankov et al., 2019; Xia et al., 2020). Additionally, Zahmatkesh et al. (Zahmatkesh et al., 2022) found that exosomes derived from *H. pylori*-OMVs-infected hepatocytes activated hepatic stellate cells and upregulated the overexpression of liver fibrosis markers (vimentin, cadherin 1, and catenin beta 1). However, the role of *H. pylori*-OMVs and EVs in NAFLD needs further exploration.

## 5 Conclusion

*H. pylori* infection and NAFLD are chronic diseases, and their prolonged progression may lead to irreversible damage to the body. If *H. pylori* eradication therapy can delay or improve the physiological status of *H. pylori* positive NAFLD patients, it will significantly alleviate the disease burden of NAFLD. Numerous clinical and animal studies have suggested a link between *H. pylori* infection and NAFLD. We summarize here meta-analyses investigating the association between *H. pylori* infection and NAFLD, analyze some studies that oppose the association between them, and enumerate a number of high-quality evidence.

Understanding the relationship between *H. pylori* infection and NAFLD contributes to comprehending the mechanisms by which *H. pylori* leads to extragastric disease. This comprehension helps clinicians better understand and manage NAFLD and facilitates NAFLD patients with *H. pylori* infection to benefit from *H. pylori* eradication. Future multicenter prospective studies are needed to illustrate how *H. pylori* eradication will improve the physiological status of NAFLD patients or whether *H. pylori* eradication has additional benefits for NAFLD patients. In experimental studies, it is critical to determine whether *H. pylori* infection affects the progression of NAFLD in a direct (e.g., EVs or *H. pylori*-OMVs) or indirect (e.g., systemic chronic low-grade inflammation, IR) manner.

## Author contributions

XC: Writing – original draft, Conceptualization, Data curation, Visualization. RP: Writing – original draft, Data curation, Visualization. DP: Writing – original draft, Data curation, Visualization. JX: Writing – original draft, Data curation, Visualization. DL: Writing – review & editing, Methodology, Supervision. RL: Writing – review & editing, Methodology, Supervision.
